# Supplementary material for: Gastric Cancer Risk in Association with Underweight, Overweight, and Obesity: A Systematic Review and Meta-Analysis
Source: Cancers (Basel). 2023 May 16;15(10):2778. doi: 10.3390/cancers15102778 (PMC10216434; doi:10.3390/cancers15102778)
Supplement: Supplementary file 1 [file cancers-15-02778-s001.zip › cancers-2275478-supplementary.pdf]

This is supplementary file to "**Gastric Cancer Risk in Association with Underweight, Overweight, and Obesity: A Systematic Review and Meta-Analysis**" by *Narges Azizi, Moein Zangiabadian, Golnoosh Seifi, Afshan Davari, Elham Yekekhani, Seyed Amir Ahmad Safavi-Naini, Nathan A. Berger, Mohammad Javad Nasiri* (mj.nasiri@hotmail.com) *, Mohammad Reza Sohrabi* (m.sohrabi@sbmu.ac.ir*).*

Table of content

Table S1: Search strategy used for Embase, Pubmed/Medline and Scopus (Date: 1.1.2000 till 1.1.2023).

Table S2: Study characteristics and multivariate adjustment results in full detail (summary of this table is presented in **Table 1** of the manuscript).

Table S3. Subgroup analysis of race, and gender effect on gastric cardia cancer in different body mass index categories.

Table S4. Subgroup analysis of race, and gender effect on gastric non-cardia cancer in different body mass index categories.

Table S5. Previous meta-analysis exploring association of body mass index (BMI) and gastric cancer.

Figure S1. The country map of included cohort studies.

Figure S2. One-leave-out analysis

Figure S3. Funnel plot of included studies showing effect of different body mass index groups on gastric cancer risk.

Figure S4. The relative risk graph used in visualization of graphical abstract.

Table S1. Search strategy used for Embase, Pubmed/Medline and Scopus (Date: 1.1.2000 till 1.1.2023)

**Embase Database (A and B); Date:** 1.1.2000 till 1.1.2023	
A. Gastric cancer	B. Risk factors
Stomach tumor	Risk factor or<br>Body weight or<br>Obesity or<br>Lifestyle or<br>Demography or<br>Social aspect or<br>Socioeconomics or<br>Sociodemographics or<br>Metabolic syndrome x
**Search strategy and number of retrieved articles:**<br>1: ('stomach tumor'/exp OR 'stomach tumor') AND ('body mass'/exp OR 'body mass') AND ('risk factor'/exp OR 'risk factor') AND [2000-2023]/py<br>No:800<br>2: ('stomach tumor'/exp OR 'stomach tumor') AND ('body weight'/exp OR 'body weight') AND ('risk factor'/exp OR 'risk factor') AND [2000-2023]/py<br>No:401<br>3: ('stomach tumor'/exp OR 'stomach tumor') AND ('obesity'/exp OR obesity) AND ('risk factor'/exp OR 'risk factor') AND [2000-2023]/py<br>No:619<br>4: ('stomach tumor'/exp OR 'stomach tumor') AND ('lifestyle'/exp OR lifestyle) AND ('risk factor'/exp OR 'risk factor') AND [2000-2023]/py<br>No:422<br>5: ('stomach tumor'/exp OR 'stomach tumor') AND ('demography'/exp OR demography) AND ('risk factor'/exp OR 'risk factor') AND [2000-2023]/py<br>No:266<br>6: ('stomach tumor'/exp OR 'stomach tumor') AND ('social aspect'/exp OR 'social aspect') AND [2000-2023]/py<br>No:67<br>7: ('stomach tumor'/exp OR 'stomach tumor') AND ('socioeconomics'/exp OR socioeconomics) AND [2000-2023]/py<br>No:4291<br>8: ('stomach tumor'/exp OR 'stomach tumor') AND ('sociodemographics'/exp OR sociodemographics) AND [2000-2023]/py<br>No:48<br>9: ('stomach tumor'/exp OR 'stomach tumor') AND ('risk factor'/exp OR 'risk factor') AND [2000-2023]/py<br>No:10455<br>10: ('stomach tumor'/exp OR 'stomach tumor') AND ('risk factor'/exp OR 'risk factor') AND ('metabolic syndrome x'/exp OR 'metabolic syndrome x') AND [2000-2023]/py<br>No:64	
**Pubmed/Medline (Criteria A and B and C); Date:** 1.1.2000 till 1.1.2023	

A. Gastric cancer	B. Risk factor	C. Filters
Stomach neoplasms	Risk Factors or<br>Life Style or<br>Demography or<br>Social Factors or<br>Socioeconomic Factors or<br>Sociodemographic Factors or<br>Body Mass Index or<br>Body Weight or<br>Obesity or<br>Metabolic Syndrome	Observational Study

Search strategy and number of retrieved articles:
1: (("Stomach Neoplasms"[Mesh]) AND "Body Mass Index"[Mesh]) AND "Risk Factors"[Mesh]
No: 161
2: (("Stomach Neoplasms"[Mesh]) AND "Body Weight"[Mesh]) AND "Risk Factors"[Mesh]
No: 148
3: (("Stomach Neoplasms"[Mesh]) AND "Obesity"[Mesh]) AND "Risk Factors"[Mesh]
No: 98
4: (("Stomach Neoplasms"[Mesh]) AND "Life Style"[Mesh]) AND "Risk Factors"[Mesh]
No: 106
5: (("Stomach Neoplasms"[Mesh]) AND "Demography"[Mesh]) AND "Risk Factors"[Mesh]
No: 1457
6: ("Stomach Neoplasms"[Mesh]) AND "Social Factors"[Mesh]
No:0
7: ("Stomach Neoplasms"[Mesh]) AND "Socioeconomic Factors"[Mesh]
No: 276
8: ("Stomach Neoplasms"[Mesh]) AND "Sociodemographic Factors"[Mesh]
No:1
9: ("Stomach Neoplasms"[Mesh]) AND "Risk Factors"[Mesh]
No: 5038
10: (("Metabolic Syndrome"[Mesh]) AND "Stomach Neoplasms"[Mesh]) AND "Risk Factors"[Mesh]
No:11

Scopus (Criteria A and B); Date: 1.1.2000 till 1.1.2023

**A**. Gastric cancer	B. Risk factor
Stomach cancer or<br>Stomach neoplasms or<br>Gastric cancer or<br>Gastric neoplasms<br>or Gastrointestinal neoplasms	Risk Factors or<br>Demographic factors or<br>Obesity or<br>Sociodemographic Factors or<br>Body Mass Index or<br>Body Weight

Search strategy and number of retrieved articles:
(TITLE-ABS-KEY (stomach AND cancer) OR TITLE-ABS-KEY (stomach AND neoplasms) OR TITLE-ABS-KEY (gastric AND cancer) OR TITLE-ABS-KEY (gastric AND neoplasms) OR TITLE-ABS-KEY (gastrointestinal AND neoplasms) AND TITLE-ABS-KEY (risk AND factors) OR TITLE-ABS-KEY (demographic AND factors) OR TITLE-ABS-KEY (obesity) OR TITLE-ABS-KEY (sociodemographic AND factors) OR TITLE-ABS-KEY (body AND mass AND index) OR TITLE-ABS-KEY (body AND weight)

AND NOT TITLE-ABS-KEY (surgery) AND NOT TITLE-ABS-KEY (treatment) AND NOT TITLE-ABS-KEY (survival) AND NOT TITLE-ABS-KEY (mortality) AND NOT TITLE-ABS-KEY (systematic AND review) AND NOT TITLE-ABS-KEY (meta AND analysis) AND NOT TITLE-ABS-KEY (nonhuman) AND NOT TITLE-ABS-KEY (case AND report) AND NOT TITLE-ABS-KEY (cross-sectional) AND NOT TITLE-ABS-KEY (case AND control AND study)) AND PUBYEAR > 1999 AND PUBYEAR < 2023 AND PUBYEAR > 1999 AND PUBYEAR < 2023
No:4647

Table S2. Study characteristics and multivariate adjustment results in full detail (summary of this table is presented in **Table 1** of the manuscript)

Study, Year, Location	Type of study; Race	Outcome	Ascertainment of exposure	Adjustments	Effect size measure	Result of multivariate adjusted analysis HR (95 %CI)
Rapp et al, 2005, Austria (55)	Prospective Cohort Study; Non-Asian	Gastric Adenocarcinoma	Measured by trained personnel	Stratified according to sex and age at enrolment and adjusted for smoking status and occupational groups.	HR (95 %CI)	GC: Men: Overweight: 1.04 (0.73-1.47), obesity: 0.72 (0.40-1.33)<br>Women: Overweight: 0.78 (0.51-1.20), obesity: 1.28 (0.76-2.15)
Lukanova et al, 2006, Japan (56)	Prospective Cohort Study; Non-Asian	Gastric Adenocarcinoma	Measured by trained personnel	Adjusted for age, calendar year, and smoking.	RR (95 %CI)	GC: Men: Overweight 1.36 (0.75–2.57)<br>Women: Overweight: 0.53 (0.22–1.18)
Sjödahl et al, 2008, Norway (57)	Prospective Cohort Study; Non-Asian	Gastric Adenocarcinoma, Gastric Cardia Adenocarcinoma, Gastric Noncardia Adenocarcinoma	Measured by trained personnel	Adjustments were made to attain age, gender, smoking status (never, former, and current), alcohol use (frequency during the last two weeks: never, not been drinking, 1-4 times, and z5 times), salt intake (consumption of highly salted foods: 0-1 time per	HR (95 %CI)	GC: Underweight: 0.7 (0.1-5.2); overweight: 1.0 (0.7-1.4), Obesity: 1.1 (0.7-1.8)<br>GNCC: Underweight: 0.9 (0.1-6.7); overweight: 1.1 (0.7-1.6), obesity: 1.2 (0.7-2.1)

				month,<br>>1-2 times per month, up to once per week, and z2 times per week), and occupation (higher-level employees or employers and professionals, intermediate employees, lower-level employees, other self-employed or farmers, skilled laborers, and unskilled laborers).		
Abnet et al, 2008, Six USA states * (58)	Prospective Cohort Study; Non-Asian	Gastric Adenocarcinoma, Gastric Cardia Adenocarcinoma, Gastric Noncardia Adenocarcinoma	A self-reported questionnaire	Adjusted for age, sex, cigarette smoking, alcohol consumption, education, physical activity, and race.	HR (95 %CI)	GCC: Underweight: 0.70 (0.10–5.06), overweight: 1.06 (0.79–1.41), Obese: 1.70 (1.22–2.36);<br>GNCC: Underweight: 2.97 (1.38–6.39), overweight: 0.80 (0.61–1.04), Obese: 1.08 (0.78–1.50)
Eom et al, 2015, Korea (59)	Prospective Cohort Study; Asian	Gastric Adenocarcinoma	Measured by trained personnel	Crude and age (at baseline) adjusted analyses were performed separately for men and women.	HR (95 %CI)	GC: Men: Underweight: 1.135 (1.051-1.226), overweight: 0.895 (0.864-0.927);<br>Women GC: Underweight: 1.160 (1.010-1.333), overweight: 0.966 (0.906-1.030)

Sanikini et al, 2019, ten European countries ** (60)	Prospective Cohort Study; Non-Asian	Gastric Adenocarcinoma, Gastric Cardia Adenocarcinoma, Gastric Noncardia Adenocarcinoma	Measured by trained personnel	Stratified on age, study center and adjusted for smoking, education level and alcohol intake.	HR (95 %CI)	GCC: Men: Overweight: 1.22 (0.86–1.75), obese: 0.94 (0.55–1.61);<br>Women: Overweigh: 1.44 (0.85–2.43), obesity: 1.41 (0.70–2.83);<br>GNCC: Men: Overweight: 1.13 (0.79–1.62); 1.03 (0.64–1.65);<br>Women: Overweight: 0.96 (0.67–1.38), obesity: 1.31 (0.86–2.00)
Sanikini et al, 2020, UK (61)	Prospective Cohort Study; Non-Asian	Gastric Adenocarcinoma, Gastric Cardia Adenocarcinoma, Gastric Noncardia Adenocarcinoma	Measured by trained personnel	Stratified on age (5-year categories), sex, Townsend deprivation index (quintiles), recruitment assessment center and adjusted for smoking status and education.	HR (95 %CI)	GCC: Overweight: 1.13 (0.71–1.82); obesity: 1.32 (0.79–2.21);<br>GNCC: Overweight: 0.74 (0.45–1.23), obesity: 0.74 (0.42–1.32)
Zhang et al, 2020, China (53)	Prospective Cohort Study; Asian	Gastric Adenocarcinoma	Measured by trained personnel	Adjusted for age at baseline, gender, smoking, drinking, family history of cancer, education, and consumption of fresh fruit.	HR (95 %CI)	GC: Underweight: 0.99(0.78–1.26), overweight or obesity: 1.06(0.73–1.55)

Wang et al, 2020 China (62)	Retrospective Cohort Study; Asian	Gastric Adenocarcinoma	Measured by trained personal	Adjusted HRs were calculated with the use of age as the underlying time scale, stratified according to age at risk (in 5-year intervals), sex and region (10 study sites), adjusted for level of education (no formal school, primary school, middle school, high school, college or university or higher), marital status (married, widowed, divorced, separated or never married), annual household income (<10,000 Yuan, 10,000–19,999 Yuan, 20,000–34,999 Yuan or ≥35,000 Yuan), alcohol consumption (nondrinker, occasional drinker, former drinker, or current regular drinker), smoking status (never smoker, occasional smoker, former	HR (95 %CI)	GC: Underweight: 1.47 (1.22, 1.77), overweight: 0.94 (0.85, 1.06), obesity: 0.95 (0.76, 1.20)

				smoker or current regular smoker), physical activity (metabolic equivalent of task [MET] hr/day).		
Choi et al., 2021, Korea (63)	Prospective Cohort Study; Asian	Gastric Adenocarcinoma	Measured by trained personal	Adjusted for smoking, alcohol consumption, regular exercise, income, age at menarche, parity, duration of breastfeeding, duration of oral contraceptive use in premenopausal women and duration of hormone replacement therapy and age at menopause in postmenopausal women.	HR (95 %CI)	GC in premenopausal women: Underweight: 1.12 (0.95-1.33), overweight: 0.96 (0.89-1.04), and obesity: 1.02 (0.94- 1.11)<br>GC in postmenopausal women: Underweight: 1.07 (1.00-1.14), overweight: 1.01 (0.99-1.04), and obesity: 1.03 (1.00- 1.05)
Lee et al., 2022, Korea (64)	Prospective Cohort Study; Asian	Gastric Adenocarcinoma	Measured by trained personal	Adjusted for sex, education, smoking status, drinking status, family history of gastric cancer, exercise and total energy intake.	HR (95 %CI)	GC (BMI at Baseline survey): Underweight: 0.67 (0.36-1.26), overweight 0.95 (0.81-1.11) and obesity: 1.08 (0.93-1.25)

				Categorized for different age groups.		
Lim et al., 2022, Korea (65)	Prospective Cohort Study; Asian	Gastric Adenocarcinoma	Measured by trained personal	Adjusted for age, sex, smoking, alcohol consumption, regular exercise, income, diabetes, hypertension, and dyslipidemia.	HR (95 %CI)	GC: Underweight: 1.15 (1.03-1.29), overweight 0.98 (0.93-1.02) and obesity: 1.03 (0.98-1.07)
Jang et al., 2022, thirteen cohorts from four Asian countries *** (54)	Prospective Cohort Study; Asian	Gastric Adenocarcinoma, Gastric Cardia Adenocarcinoma, Gastric Noncardia Adenocarcinoma	Measured by trained personal	Adjusted for age (at enrollment), sex, country, cohort, smoking status, and alcohol drinking status.	HR (95 %CI)	GC: Underweight: 1.15 (1.05–1.25), overweight: 1.01 (0.94–1.08), Obese: 1.12 (1.03–1.22); GCC: Underweight: 0.89 (0.58–1.38), overweight: 1.16 (0.86–1.57), Obese: 0.94 (0.62–1.43); GNCC: Underweight: 1.22 (1.10–1.35), overweight: 0.97 (0.89–1.05), Obese: 1.09 (0.98–1.21)

Footnote: * six U.S. states including California, Florida, Louisiana, New Jersey, North Carolina, and Pennsylvania) and two metropolitan areas (Atlanta, Georgia, and Detroit, Michigan. **Ten European countries including Denmark, France, Germany, Greece, Italy, Norway, Spain, Sweden, the Netherlands and the United Kingdom. *** Four Asian countries including China, Japan, Korea, and Singapore. Abbreviations: GC; Gastric Cancer, GCC, gastric cardia cancer; GNCC, gastric non-cardia cancer; HR, hazard ratio; CI, confidence interval.

Table S3. Subgroup analysis of race, and gender effect on gastric cardia cancer in different body mass index categories.

Potential factors	RR (CI 95%)	No of Studies	Heterogeneity χ^2	P value	I^2%	Interaction P value
**Obesity**						
Race	Subgroup analysis					
Asian<br>Non-Asian	0.750(0.595-0.946)<br>1.641(1.207-2.232)	1<br>4	-<br>5.39	-<br>0.145	-<br>44.38	-
Gender	Subset analysis					
Male<br>Female	1.357(0.605-3.042)<br>0.990(0.396-2.475)	2	4.48<br>2.05	0.034<br>0.152	77.69<br>51.20	0.613
All studies	1.318(0.803-2.162)	5	32.52	0.000	87.70	-
**Overweight**						
Race	Subgroup analysis					
Asian<br>Non-Asian	0.774(0.612-0.979)<br>1.541(1.294-1.834)	1<br>4	-<br>2.54	-<br>0.469	-<br>0.00	-
Gender	Subset analysis					
Male<br>Female	1.334(0.999-1.781)<br>1.190(0.510-2.777)	2	0.15<br>2.80	0.701<br>0.094	0.00<br>64.28	0.803
All studies	1.292(0.899-1.858)	5	23.77	0.000	83.17	-
**Underweight**						
Race	Subgroup analysis					
Asian<br>Non-Asian	0.956(0.641-1.424)<br>0.972(0.360-2.623)	1<br>4	-<br>1.69	-<br>0.639	-<br>0.00	-
Gender	Subset analysis					
Male	3.162(0.772-12.952)	2	0.72	0.395	0.00	0.356

Female	1.004(0.138-7.327)		0.50	0.482	0.00	
All studies	0.958(0.662-1.387)	5	1.69	0.792	0.00	-

Table S4. Subgroup analysis of race, and gender effect on gastric non-cardia cancer in different body mass index categories.

Potential factors	RR (CI 95%)	No of Studies	Heterogeneity χ^2	P value	I²%	Interaction P value
**Obesity**						
Race	Subgroup analysis					
Asian<br>Non-Asian	0.685(0.642-0.730)<br>1.406(1.061-1.864)	1<br>4	-<br>7.33	-<br>0.062	-<br>59.05	-
Gender	Subset analysis					
Male<br>Female	1.344(0.918-1.969)<br>1.663(0.858-3.222)	2	0.97<br>2.25	0.325<br>0.134	0.00<br>55.56	0.585
All studies	1.182(0.728-1.919)	5	69.61	0.000	94.25	-
**Overweight**						
Race	Subgroup analysis					
Asian<br>Non-Asian	0.754(0.707-0.803)<br>1.248(0.930-1.675)	1<br>4	-<br>12.14	-<br>0.007	-<br>75.29	-
Gender	Subset analysis					
Male<br>Female	1.315(0.915-1.889)<br>1.246(0.745-2.083)	2	1.24<br>1.70	0.266<br>0.193	19.22<br>40.97	0.866
All studies	1.107(0.765-1.602)	5	59.18	0.000	93.24	-
**Underweight**						

Race	Subgroup analysis					
Asian	1.388(1.268-1.520)	1	-	-	-	-
Non-Asian	1.756(0.763-4.044)	4	6.342	0.096	52.70	
Gender	Subset analysis					
Male	5.809(0.729-46.275)	2	2.85	0.091	64.93	0.154
Female	0.995(0.283-3.501)		0.20	0.651	0.00	
All studies	1.611(0.975-2.660)	5	7.78	0.100	48.60	-

Table S5. Previous meta-analysis exploring association of body mass index (BMI) and gastric cancer (1)

First author, year	Included studies, (N)	Meta-analysis	BMI categories	Cancer	Subgroup (N)	Underweight	Overweight	Obesity	Note
Turati, 2013 (31)	Case-control and cohort, (N= 22)	Random effect, Estimate of OR in case-control and HR in cohort studies. Dose–risk analysis.	WHO; when one category fell in range, they combined the corresponding estimates	Esophageal adenocarcinoma (EA)and cardiaia adenocarcinoma (GCA)	Total<br>Male (10)<br>Female (8)<br>Asia (68)<br>GCA (13)<br>EA (17)	-	1.71 (1.50–1.96)<br>2.13 (1.63–2.78)<br>1.59 (1.20–2.09)<br>2.44 (1.01–5.88)<br>1.40 (1.18–1.66)<br>1.87 (1.61–2.17)	2.34 (1.95–2.81)<br>2.17 (1.56–3.01)<br>2.28 (1.64–3.18)<br>2.10 (0.84–5.23)<br>1.93 (1.52–2.45)<br>2.73 (2.16–3.46)	I: The analysis is performed on both EA and GCA cancer, and stratified analysis is not available.<br>II: No formal statistical approach for comparison of subgroups
Chen, 2013 (29)	Prospective studies (N= 24)	Random effect, summary relative risk (SRR); Dose-response analysis; Stratified meta-analysis; Subgroup analysis	WHO (but also include studies with other BMI categories)	GC	Total<br>Cardia GC<br>Non-cardia<br>Male (10)<br>Female (9)<br>Asia (68)<br>Non-Asia (11)	-	1.01 (0.96-1.07)<br>1.21 (1.03-1.42)<br>0.93 (0.82-1.05)<br>1.07 (1.01–1.03)<br>0.99 (0.89–1.11)<br>1.06 (1.00–1.12)<br>0.98 (0.92–1.04)	1.06 (0.99-1.12)<br>1.82 (1.32-2.49)<br>1.00 (0.87-1.15)<br>1.12 (1.00–1.24)<br>1.04 (0.93–1.16)<br>1.06 (0.99–1.14)<br>1.01 (0.84–1.21)	I: although the sought to include WHO BMI categories, other categories are also included<br>II: the input of meta-analysis (RR) had been adjusted for different confounders among studies; and as they stated in subgroup analysis, different

									adjustments resulted in different effects among studies<br>III: They stated pooled RR is “somewhat higher for males” while the confidence interval range suggest no gender difference (No formal statistical approach for comparison of subgroups).
Lin, 2014 (28)	Cohort and case-control (N= 16); excluded duplicate populations	Adjusted RR analyzed from RR, HR or OR of GC incidence at least adjusted for age; Subgroup analysis	Both WHO and Chinese classification; Strict reference range (BMI= 18.5 to 25 kg/m2)	GC	Total<br>Male (5)<br>Female (68)<br>Cardia (7)<br>Non-cardia (68)<br>Asia (68)<br>Non-Asia (12)	-	1.04 (0.96-1.12)<br>1.12 (0.96– 1.29)<br>0.87 (0.71– 1.05)<br>1.22 (1.05– 1.42)<br>0.94 (0.81– 1.10)<br>1.41 (0.93– 2.14)<br>0.98 (0.92– 1.04)	1.13 (1.03-1.24)<br>1.27 (1.09–1.48)<br>1.04 (0.79–1.39)<br>1.61 (1.15–2.24)<br>0.83 (0.68–1.01)<br>1.11 (0.82–1.50)<br>1.14 (1.02–1.28)	I: No formal statistical approach for comparison of subgroups
Renehnan, 2008 (26)	Prospective observational			All cancer types including GC	Male (8)<br>Female (5)	-	RR per 5kg/m2:<br>1·03 (1·00–1·07)<br>1·04 (0·90–1·20)		
Yang, 2009 (27)	Cohort (N= 10)		WHO	Gastric cancer	Total (7)<br>Male (68)<br>Female (68)		1.21 (1.08–1.36)<br>1.10 (1.03–1.18)<br>1.12 (0.90–1.40)	1.36 (1.21–1.54)<br>1.41 (1.08–1.83)<br>1.16 (0.89–1.51)	I: No formal statistical approach for comparison of subgroups

					Cardia (4)<br>Non-cardia (4)<br>Asian (4)<br>Non-Asian (68)		1.40 (1.16–1.68)<br>1.16 (0.94–1.43)<br>1.17 (0.88–1.56)<br>1.24 (1.14–1.36)	2.06 (1.63–2.61)<br>1.26 (0.89–1.78)<br>-<br>-	
Bae, 2020 (30)	Asian Population based Cohort (N=7)	logarithmic RR from RR of included studies	Asia-Pacific Classification	Gastric cancer	Asian Men<br>Asian Women		-<br>0.99 (0.74-1.32	0.84 (0.73-0.97)<br>1.08 (0.72-1.63)	I: lack of strong search methodology<br>II: single authored data extraction and high risk of error

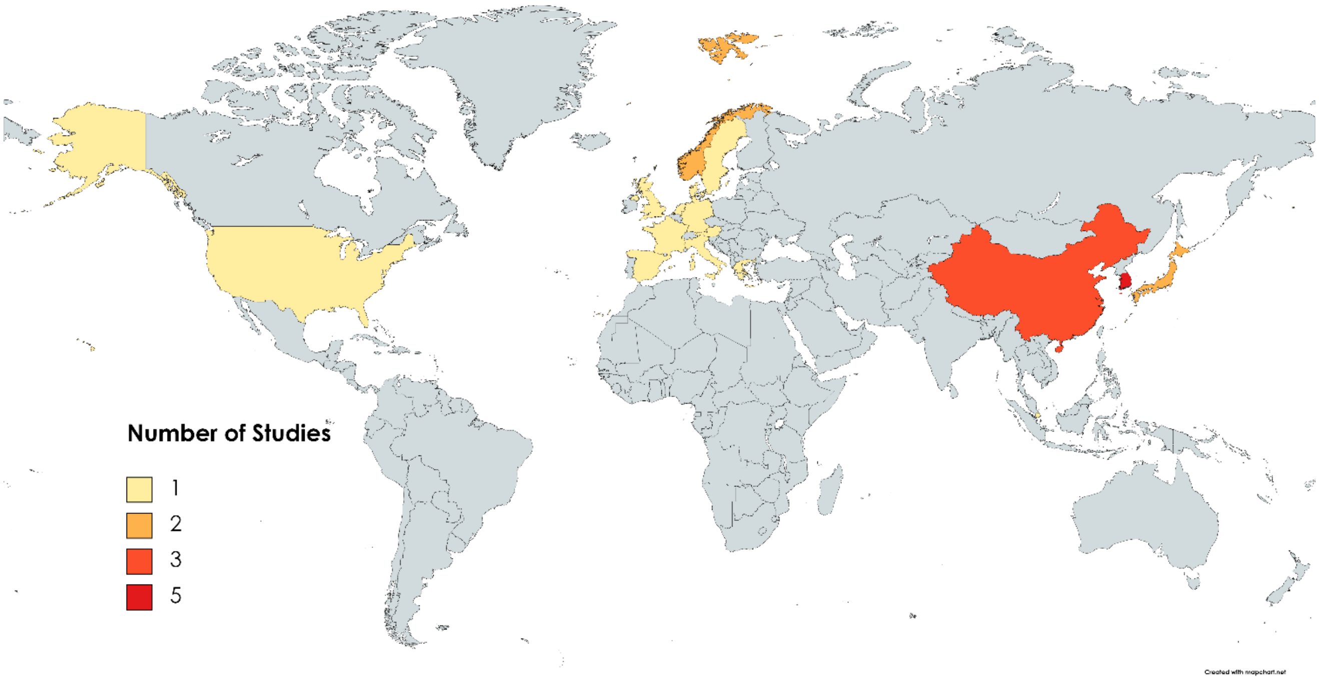

Figure S1. The country map of included cohort studies.

Footnote: One study performed analysis on multiple cohorts (based on thirteen cohorts from China (two), Japan (eight), Korea (two), and Singapore (one)). In the picture, we added one point to each country included in this study.

Obesity

Author	Year	Point	Lower limit	Upper limit	p-Value
Rapp	2005	1.185	1.037	1.354	0.012
Jang	2022	1.275	1.138	1.429	0.000
Lee	2022	1.204	1.050	1.381	0.008
Sjodahl	2008	1.206	1.054	1.378	0.006
Abnet	2008	1.190	1.039	1.362	0.012
Eom	2015	1.261	1.087	1.462	0.002
Sanikini	2019	1.185	1.036	1.356	0.013
Sanikini	2020	1.202	1.051	1.374	0.007
Wang	2020	1.251	1.092	1.433	0.001
Choi	2021	1.216	1.055	1.403	0.007
Lim	2022	1.230	1.055	1.436	0.008
		1.218	1.070	1.386	0.003

(Statistics with study removed)

Risk ratio (95% CI) with study removed

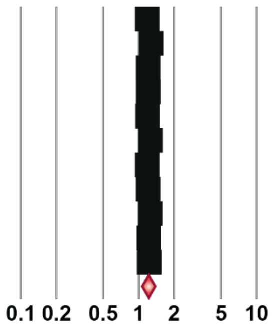

Overweight

Author	Year	Point	Lower limit	Upper limit	p-Value
Rapp	2005	1.124	1.022	1.237	0.016
Lee	2022	1.157	1.047	1.278	0.004
Lim	2022	1.156	1.040	1.284	0.007
Jang	2022	1.188	1.096	1.289	0.000
Lukanova	2006	1.145	1.040	1.260	0.006
Sjodahl	2008	1.140	1.034	1.256	0.009
Abnet	2008	1.160	1.051	1.282	0.003
Eom	2015	1.190	1.059	1.337	0.003
Sanikini	2019	1.111	1.011	1.221	0.028
Sanikini	2020	1.149	1.043	1.266	0.005
Wang	2020	1.186	1.073	1.311	0.001
Choi	2021	1.174	1.042	1.323	0.008
		1.155	1.051	1.270	0.003

(Statistics with study removed)

Risk ratio (95% CI) with study removed

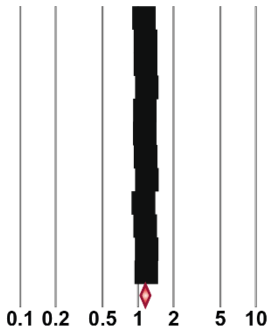

Underweight

Author	Year	Point	Lower limit	Upper limit	p-Value
Sjodahl	2008	1.129	0.971	1.313	0.115
Jang	2022	1.095	0.917	1.309	0.316
Lee	2022	1.152	0.991	1.339	0.066
Abnet	2008	1.102	0.947	1.281	0.210
Eom	2015	1.104	0.913	1.336	0.307
Sanikini	2019	1.133	0.975	1.318	0.103
Sanikini	2020	1.106	0.953	1.283	0.185
Zhang	2020	1.144	0.975	1.344	0.100
Wang	2020	1.078	0.919	1.264	0.358
Choi	2021	1.115	0.915	1.360	0.281
Lim	2022	1.227	1.125	1.338	0.000
		1.124	0.968	1.304	0.125

(Statistics with study removed)

Risk ratio (95% CI) with study removed

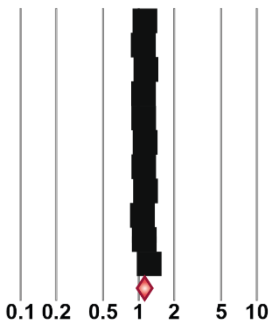

Figure S2. One-leave-out analysis

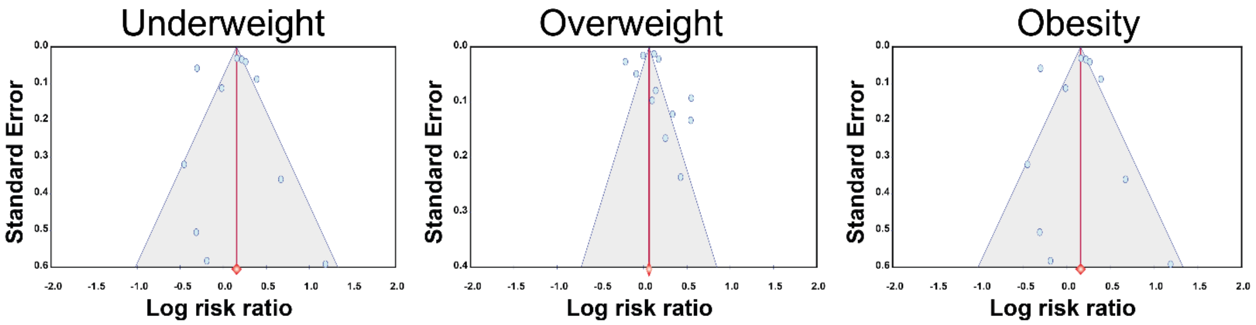

Figure S3. Funnel plot of included studies showing effect of different body mass index groups on gastric cancer risk.

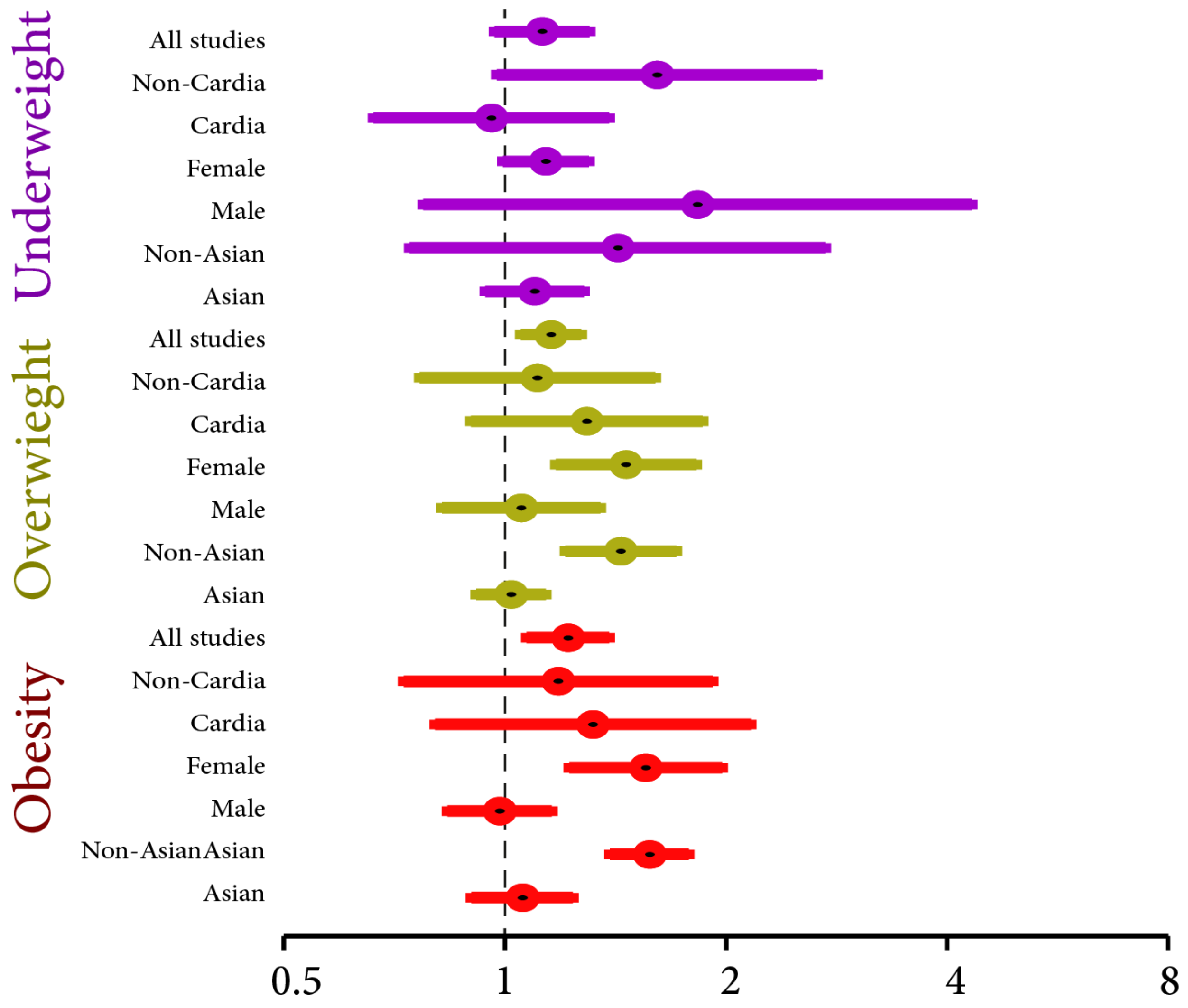

Figure S4. The relative risk graph used in visualization of graphical abstract.
